# Analyzing siRNA Concentration, Complexation and Stability in Cationic Dendriplexes by Stem-Loop Reverse Transcription-qPCR

**DOI:** 10.3390/pharmaceutics14071348

**Published:** 2022-06-25

**Authors:** Maximilian Neugebauer, Clara E. Grundmann, Michael Lehnert, Felix von Stetten, Susanna M. Früh, Regine Süss

**Affiliations:** 1 Hahn-Schickard, Georges-Koehler-Allee 103, 79110 Freiburg, Germany; michael.lehnert@hahn-schickard.de (M.L.); felix.von.stetten@hahn-schickard.de (F.v.S.); susanna.frueh@imtek.uni-freiburg.de (S.M.F.); 2 Laboratory for MEMS Applications, IMTEK—Department of Microsystems Engineering, University of Freiburg, Georges-Koehler-Allee 103, 79110 Freiburg, Germany; 3 Department of Pharmaceutical Technology and Biopharmacy, Institute of Pharmaceutical Sciences, University of Freiburg, Sonnenstr. 5, 79104 Freiburg, Germany; cgrundmann@pharmazeutischetechnologie.de (C.E.G.); regine.suess@pharmazie.uni-freiburg.de (R.S.)

**Keywords:** reverse transcription real-time polymerase chain reaction (RT-qPCR), stem-loop primer, small interfering RNA (siRNA), drug delivery, dendriplexes, nitrogen-to-phosphate (N/P) ratio, ribonuclease A (RNase A) digest

## Abstract

RNA interference (RNAi) is a powerful therapeutic approach for messenger RNA (mRNA) level regulation in human cells. RNAi can be triggered by small interfering RNAs (siRNAs) which are delivered by non-viral carriers, e.g., dendriplexes. siRNA quantification inside carriers is essential in drug delivery system development. However, current siRNA measuring methods either are not very sensitive, only semi-quantitative or not specific towards intact target siRNA sequences. We present a novel reverse transcription real-time PCR (RT-qPCR)-based application for siRNA quantification in drug formulations. It enables specific and highly sensitive quantification of released, uncomplexed target siRNA and thus also indirect assessment of siRNA stability and concentration inside dendriplexes. We show that comparison with a dilution series allows for siRNA quantification, exclusively measuring intact target sequences. The limit of detection (LOD) was 4.2 pM (±0.2 pM) and the limit of quantification (LOQ) 77.8 pM (±13.4 pM) for uncomplexed siRNA. LOD and LOQ of dendriplex samples were 31.6 pM (±0 pM) and 44.4 pM (±9.0 pM), respectively. Unspecific non-target siRNA sequences did not decrease quantification accuracy when present in samples. As an example of use, we assessed siRNA complexation inside dendriplexes with varying nitrogen-to-phosphate ratios. Further, protection of siRNA inside dendriplexes from RNase A degradation was quantitatively compared to degradation of uncomplexed siRNA. This novel application for quantification of siRNA in drug delivery systems is an important tool for the development of new siRNA-based drugs and quality checks including drug stability measurements.

## 1. Introduction

**Figure 1:**
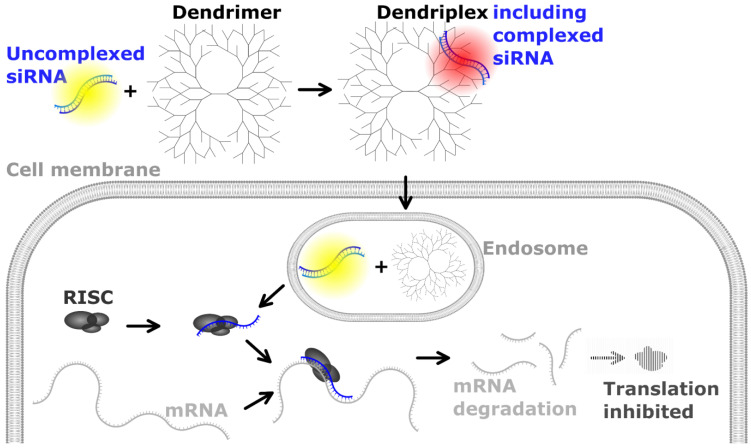
Gene silencing via small interfering RNA (siRNA)-based drugs and current detection capabilities. siRNA binds to a carrier (e.g., dendrimer) and forms a dendriplex which enters the cell through endocytosis [19,20]. After endosomal escape, dendrimers release the siRNA and are either degraded or accumulate inside the cell [21]. Cellular RNA-induced silencing complex (RISC) formation and binding as well as processing of the siRNA to isolate its antisense strand (also known as guide strand) initiate gene silencing [22]: messenger RNA (mRNA) with reverse complementary sequence to the siRNA is degraded and hence, translation of mRNA into protein sequence inhibited. Yellow dots: uncomplexed siRNA which can be quantified using RT-qPCR as described by Chen et al. [23]. Red dot: complexed siRNA, which can indirectly be quantified in the presence of dendrimer carriers via RT-qPCR, as presented in this publication.

Small interfering RNA (siRNA) as an active substance in therapeutics has seen a sharp increase in relevance for the pharmaceutical industry in recent years [1]. Since the successful market entry of the first U.S. Food and Drug Administration (FDA)-approved siRNA-based drug Patisiran [2] in 2018, already two other siRNA therapeutics candidates have reached FDA approval status and several more are currently waiting in the pipeline at Phase III clinical stage [3]. However, transfer of this promising technology into the clinic was not achieved for a long period of time. This was mainly due to problems in establishing targeted delivery systems which enable the specific transport of a drug to specific cell types or tissues while reducing undesired systemic side effects and instability of siRNA injected into the human body [4,5,6]. Furthermore, the anionic RNA oligonucleotides are repelled by like-charged cellular membranes and hardly passively diffuse through membranes due to their size and hydrophilicity [7]. One promising approach to guide siRNA to its target cells has been followed by using dendrimers as delivery systems [8,9,10,11]. Once successfully delivered into cells, siRNA induces gene silencing processes (Figure 1) which result in modified protein biosynthesis levels. Dendrimer carriers already find applications in the treatment of a variety of diseases, such as viral infections and cancer [1,5,12,13,14]. So-called dendriplexes form spontaneously via self-assembly through electrostatic interactions between the anionic siRNA and a cationic dendrimer. Commonly used dendrimers for dendriplex formation include polyethyleneimine, poly(propyleneimine), poly-(L-lysine) or polyamidoamine (PAMAM) [15]. Dendriplexes can be modified to specifically transport the siRNA to target cells [16] as well as protecting it from degradation [17,18].

Despite the improvements made in recent years, development of siRNA delivery systems remains challenging. In case of dendrimer utilization for siRNA complexation, for example, the optimal ratio of positively charged polymer amine (N) groups to negatively charged nucleic acid phosphate (P) groups (N/P ratio) has to be defined. The N/P ratio is directly related to complexation/encapsulation efficiency [24], which has to be assessed in order to avoid over- or underdosing, highly affecting patient response [25]. N/P ratios also determine siRNA release profiles and siRNA stability since dendriplexes protect siRNA from degradation by RNase A [18,26] and siRNA is degraded much faster while still in the bloodstream than in its target site, the cytosolic environment [27]. In terms of storage and for drug quality control, siRNA stability needs to be evaluated under different conditions.

A straightforward and inexpensive way to semi-quantitatively assess siRNA in carrier complexes is via gel electrophoresis [28,29,30]. However, dyes commonly used for siRNA detection in gel electrophoresis, such as ethidium bromide [31,32] do not discriminate between target- and non-target sequences. Many other fluorescence-based siRNA quantification methods such as RiboGreen assays are susceptible to background noise [29]; they are also not sequence specific, and therefore, disputed regarding their applicability for short nucleic acid sequence measurement [33]. The detection of fluorescently [34,35], radioactively [13,36] or by other means specifically labeled siRNA does not examine the integrity of siRNA sequences and furthermore alters molecule properties and size, so that siRNA and complexes cannot be studied in their native states. Ultraviolet-visible spectroscopy measurements of unlabeled siRNA and complexes is affected by background noise in complex sample compositions, high siRNA concentrations are needed to obtain measurable absorbance values [37] and analytes are not checked for sequence identity. Several high-performance liquid chromatography based methods generate relative sequence information in comparison with known samples [38,39,40], but direct comparability between samples can be complicated, because only one sample per column is tested at a time. In summary, a technique that precisely quantifies the amount of intact target siRNA sequences inside a siRNA-dendrimer formulation and siRNA release out of the complex would be desirable in order to define and monitor the behavior of newly developed drug formulations.

**Figure 2:**
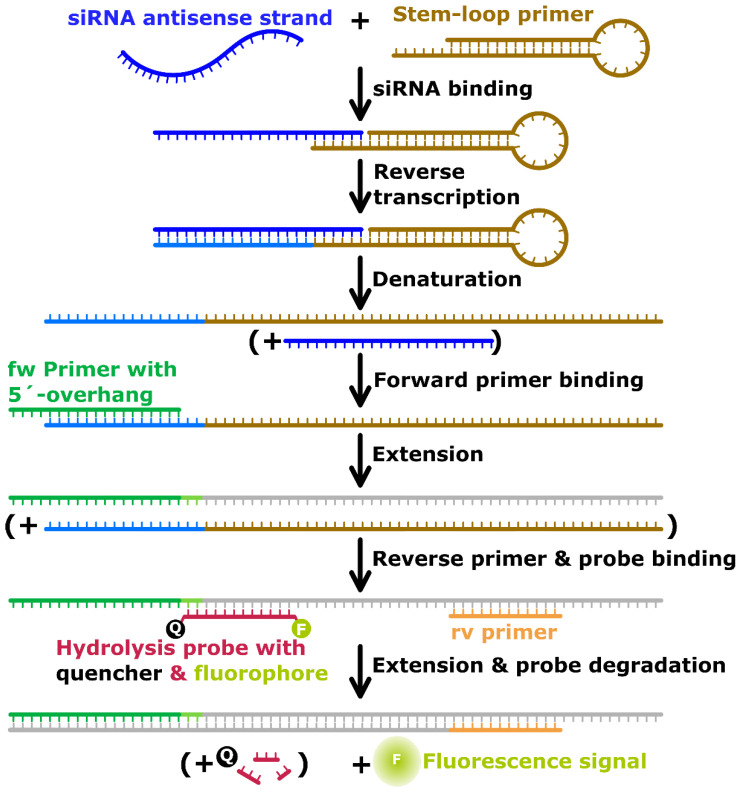
Mechanism of siRNA reverse transcription, amplification and detection via stem-loop primer and fluorescently labeled hydrolysis probes. Method adapted from Chen et al. [23] and Kramer [47]. fw primer: forward primer; rv primer: reverse primer; Q: quencher; F: fluorophore.

In this study, we present a reverse transcription real-time polymerase chain reaction (RT-qPCR)-based approach for the evaluation of siRNA inside dendriplex preparations, which does circumvent the disadvantages of the aforementioned techniques. The new assay is applicable for precise and sensitive quantification of specific siRNA sequences complexed in dendriplexes by measuring portions of uncomplexed siRNA in dendriplex samples and comparing them with a dilution series of dendriplex standard concentrations. As proof of principle, we quantified uncomplexed shares of an siRNA sequence which is able to silence the angiogenesis-inhibiting human Smad6 protein [41,42] inside samples containing mainly complexed siRNA. As dendriplexes form spontaneously via self-assembly through electrostatic interactions [43,44], preparations always contain a certain amount of uncomplexed siRNA being accessible for quantification. The dendriplexes used in this study consisted of anti-Smad6 siRNA (target siRNA) and a combination of PAMAM dendrimers and the cell penetrating peptide LAH4-L1. LAH4-L1 is a cationic, histidine-rich, amphiphilic peptide which has been successfully used in the past for the transfer of nucleic acids [45,46]. Our novel analytical application assessing dendriplexes is based on the siRNA quantification technique originally developed by Chen et al. [23] and the improved protocol by Kramer [47] (Figure 2). In this work, we added the technical possibilities to indirectly quantify the share of complexed siRNA and total siRNA in dispersions containing dendriplexes via a dendriplex dilution series under conditions preventing formation of dendriplex aggregates. We investigated whether the assay exclusively quantifies intact target siRNA sequences in their native state. Effects of other, potentially interfering nucleic acid sequences on target sequence quantification were examined. To explore the capabilities of this new application, release of siRNA from dendriplexes at different N/P ratios as well as degradation by RNase A was detected with this technique over a wide range of concentrations, down to few picomolar concentrations of siRNA. In this regard it was assessed whether the assay generally enables measurement of the stability of siRNA-based drugs as well as complexation efficiency and release profiles of siRNA complexed in dendriplex carriers.

## 2. Materials and Methods

### 2.1. Preparation of Dendriplexes

For the preparation of dendriplexes, a fixed concentration of siRNA (6.9 μM) was used, while the amount of PAMAM (generation 5, ethylenediamine core; Dendritech^®^, Midland, TX, USA) and the cell-penetrating peptide LAH4-L1 (amino acid sequence: KKALLAHALHLLALLALHLAHALKKA, provided by the Bechinger group, University of Strasbourg, France) varied according to the desired N/P ratio. Dendriplexes were freshly prepared on the day of experiment. Prior to the preparation, PAMAM and LAH4-L1 were dispersed at room temperature by vortexing in the preparation medium, called transfection-and-lyophilization-medium (TLM), which was composed of 250 mM sucrose (Caleo, Hilden, Germany) and 25 mM NaCl (Carl Roth, Karlsruhe, Germany). siRNA (Thermo Fisher Scientific Inc., Waltham, MA, USA; sequence see following subsection) was dissolved in RNase-free water (Milli-Q^®^ direct system & Biopak^®^ ultrafiltration filter by Merck KGaA, Darmstadt, Germany) at room temperature. To form the dendriplexes, TLM, PAMAM and LAH4-L1 were placed in a reaction tube and mixed by pipetting 10 times. Next, the siRNA was added and the mixture was homogenized again by repeated pipetting. Afterwards, the mixture was shaken for 20 min at 25 °C and 300 rpm in a ThermoMixer^®^ Comfort 2000 (Eppendorf, Hamburg, Germany) and finally incubated for a further 20 min at 25 °C without shaking. The dendriplexes were formed through self-assembly via electrostatic interaction of the anionic siRNA with the cationic PAMAM and LAH4-L1. The N/P ratio indicates the molar charge ratio. It is calculated from the quotient of the proportion of positively charged amino groups of the dendrimer or peptide (N = nitrogen) and the proportion of negatively charged phosphate groups of the siRNA (P = phosphate). Based on the charges per molecule, the composition of the sample can be calculated for the target N/P ratio (Equation (1)). At physiological pH, a double-stranded siRNA with 21 base pairs has 44 negative charges; the PAMAM dendrimer used carries 128 positive charges per molecule and the LAH4-L1 peptide carries five positive charges. The cationic proportion of the dendriplex formulation used in this work was composed of 10% PAMAM and 90% LAH4-L1. Dendriplex formation was confirmed by measurement of hydrodynamic diameter, polydispersity index and zeta potential which were in the range of 250–350 nm, ≤0,4 and 35–40 mV respectively for N/P = 5.

(1) $$N/P\;=\frac{n_{P\;}\times {\;N}_{P\;}^{+}+n_{L\;}\times {\;N}_{L}^{+}}{n_{s\;}\times {\;N}_{s}^{-}}$$

N/P—Nitrogen-to-phosphate ratio

$n_{P}$—Molar quantity of PAMAM (mol)

$N_{P}^{+}$—Amount of positive surface charges per PAMAM molecule

$n_{L}$—Molar quantity of LAH4-L1 (mol)

$N_{L}^{+}$—Amount of positive charges per LAH4-L1 molecule

$n_{s}$—Molar quantity of siRNA (mol)

$N_{s}^{-}$—Amount of negative charges per double stranded siRNA molecule

### 2.2. Targets, Primers and Probes

Design of stem-loop, forward and reverse primer and hydrolysis probe was carried out following general stem-loop RT-PCR structuring rules by Chen et al. [23] and according to guidelines by Kramer [47], and by using the novel software OligoPad (GNWI, Dortmund, Germany) and Visual OMP™ (DNA Software, Plymouth, MI, USA). Synthetic Ambion^®^ In Vivo anti-Smad6 siRNA templates were purchased from Thermo Fisher Scientific Inc. (Waltham, MA, USA) as target siRNA, synthetic randomized siRNA, forward and reverse primers from biomers.net GmbH (Ulm, Germany) and stem-loop primers and hydrolysis probes from Integrated DNA Technologies, Inc. (Coralville, IA, USA).

Oligonucleotide sequences are as follows (upper case letters: DNA bases; lower case letters: RNA bases; bold: locked nucleic acid; 6-FAM: 6-carboxyfluorescein; IBFQ: Iowa Black^®^ FQ; n or N: random nucleotide):

- Forward primer: 5′-GAGCAAGTCGAACACCTTGA-3′
- Reverse primer: 5′-CCAGTGCAGGGTCCGAGGTA-3′
- Stem-loop primer: 5′-GTCGTATCCAGTGCAGGGTCCGAGGTATTCGCACTGGA TACGACCTCCAT-3′
- Probe: 5′-(6-FAM)-TG**G**ATA**C**GAC**C**TCC**A**TCA-(IBFQ)-3′
- (Anti-Smad6) target siRNA sense strand: 5′-ccaucaagguguucgacuuTT-3′
- (Anti-Smad6) target siRNA antisense strand: 5′-aagucgaacaccuugauggAG-3′
- randomized siRNA: 5′-nnnnnnnnnnnnnnnnnnnNN-3′

### 2.3. Reverse Transcription-qPCR

One-step reverse transcription-qPCR (RT-qPCR) was performed in a Corbett Rotor-Gene 6000 qPCR cycler (QIAGEN, Hilden, Germany). The 10 µL RT-qPCR reactions included 5 µL qScript™ XLT One Step RT-qPCR ToughMix^®^ from Quantabio (Beverly, MA, USA), a sample volume of 1 µL and final concentrations of 1.5 µM forward primer, 0.7 µM reverse primer, 1 nM stem-loop primer and 0.2 µM hydrolysis probe. Samples without dendriplexes were also dissolved in TLM in order to ensure comparability between all samples. After preparation on ice, PCR reactions were incubated for 10 min at 50 °C for stem-loop elongation and afterwards initially denatured for 1 min at 95 °C, followed by 45 cycles of 5 s at 95 °C and 30 s at 60 °C. PCR curve fitting was accomplished with the Rotor-Gene Q Series software from QIAGEN (Hilden, Germany), using the “Dynamic Tube” function in all runs and excluding the first 5 cycles for data normalization. The fixed threshold for threshold cycle (C_t_) determination was set at 0.03 relative fluorescence units (RFU) in all experiments. All reactions were performed in triplicates.

Dilution series were carried out in 10-fold dilution steps for concentration standards containing only uncomplexed target siRNA and in $\sqrt{10}\;$≈ 3.16-fold steps for standards containing dendriplexes in order to obtain reliable standard curves in spite of a narrower dynamic range of quantification. In the latter case, dendriplex aggregation would interfere in the creation of highly reproducible dilution series when diluting the concentration standards in water or TLM medium. Because dendriplex aggregates dissolve in presence of qScript™ XLT One Step RT-qPCR ToughMix^®^, all dilution series were performed directly inside this mastermix as follows: All concentration standards (containing either dendriplexes or uncomplexed siRNA) were mixed directly with RT-qPCR ToughMix^®^, primers and probe as described above to create the first dilution step. For all other dilution steps, the previous concentration was mixed with an appropriately diluted mix of qPCR ToughMix^®^, primers, probe and TLM to ensure a constant concentration of all chemicals aside from the siRNA and dendriplex components in all reactions.

### 2.4. Preparation of siRNA Digest Using RNase A

Bovine RNase A was purchased from Thermo Fisher Scientific Inc. (Waltham, MA, USA, Cat. #: EN0531). RNase A was added in a final concentration of 1 µg/mL to the sample directly before starting incubation at 37 °C and 500 rpm in a ThermoMixer^®^ Comfort 2000 (Eppendorf, Hamburg, Germany). Digests were stopped by placing samples on ice and adding the RNase inhibitor-including qScript™ XLT One Step RT-qPCR ToughMix^®^. Immediately afterwards, the preparations for RT-qPCR analysis were performed with these samples as described in Section 2.3.

Calculation of intact siRNA percentage after incubation was done as follows: ΔC_t_ values of incubated samples were calculated by subtracting from their C_t_ values the C_t_ of non-incubated control reactions which had the same initial target siRNA concentration and were run in the same experiment. The RT-qPCR efficiency (E) was calculated by the Rotor-Gene Q Series software, measuring C_t_ shifts between different concentrations of the dilution series which was run in the same experiment. A theoretical efficiency of 2 would indicate a 100% increase in target sequences per cycle during RT-qPCR. The percentage of intact siRNA (I_%_) was then calculated using the formula:

(2) $$I_{\%}=\frac{100}{E_{\;}^{{\;\Delta C}_{t}}}$$

I_%_—Percentage of intact siRNA after incubation

E—RT-qPCR efficiency

ΔC_t_—Difference between C_t_ values of incubated samples and non-incubated controls

## 3. Results

### 3.1. RT-qPCR Assay Development

#### 3.1.1. Assay Implementation for Uncomplexed Target siRNA

**Figure 3:**
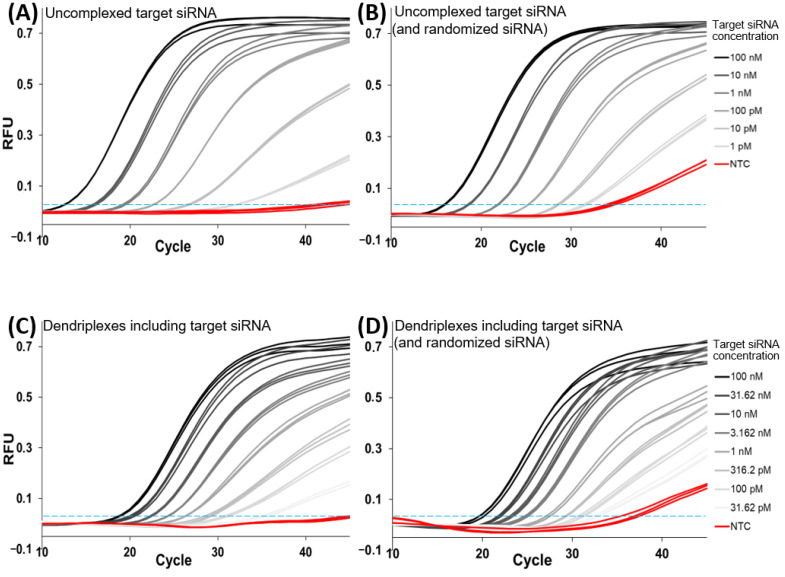
Dilution series of (anti-Smad6) target siRNA under varying conditions. Dilution series contained either only target siRNA (**A**,**C**) or additionally a constant randomized siRNA concentration of 1 µM in all reactions (**B**,**D**) and were performed either with uncomplexed target siRNA (**A**,**B**) or with dendriplexes in a constant N/P-ratio of 5 (**C**,**D**). All dilution series were performed three times in separate experiments (biological triplicates), each with technical triplicates. Indicated concentrations are final concentrations in PCR-ready samples. RFU: relative fluorescence units; NTC: no-template control; horizontal dashed lines: fixed threshold at 0.03 RFU.

**Figure 4:**
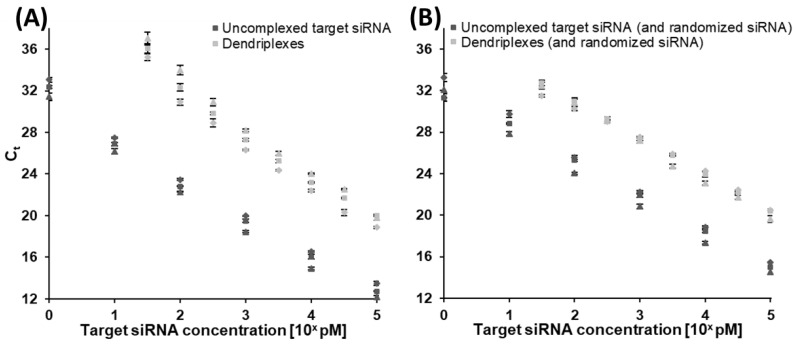
Standard curves, indicating intra and inter assay reproducibility and linearity of the RT-qPCR assay. Dilution series of anti-Smad6 (target) siRNA under varying conditions. Threshold cycle (C_t_) values of each three separate calibration curves without (**A**) or with (**B**) additionally added randomized siRNA at a constant concentration of 1 µM and with uncomplexed target siRNA or dendriplexes (constant N/P-ratio of 5 in all dilution steps, taking only target siRNA and not randomized siRNA into account). Threshold for determination of C_t_ values was kept constant at 0.03 relative fluorescence units in all experiments. All dilution series were performed three times in separate experiments (biological triplicates, represented as squares, triangles and diamonds respectively), each with technical triplicates. Arithmetic means and sample standard deviations are plotted. Indicated concentrations are final concentrations in PCR-ready samples.

An optimized set of parameters including primer sequences, buffer conditions, oligonucleotide concentrations and PCR cycling profiles for high sensitivity and, at the same time, high specificity towards the anti-Smad6 siRNA antisense strand sequence was selected (see Section 2). Using the assay, dilution series with different concentrations of double stranded anti-Smad6 siRNA (target siRNA) were tested for estimation of measurable quantification range (Figure 3A). Reproducibility was tested by performing biological triplicate reactions (meaning three independent experimental repetitions), whereas technical triplicates were used for each tested concentration. No-template controls (NTC) were also performed in triplicate and showed no amplification of fluorescence signals. The experiment was repeated adding randomized siRNA (Figure 3B) in very high concentrations (1 µM final concentration in all dilution steps) as potential PCR inhibitor and competitor of the anti-Smad6 siRNA for testing sensitivity and reproducibility changes as well as specificity. A slight increase in fluorescence values over time was observed in NTCs with added randomized siRNA and PCR curves of consecutive dilution steps were compressed (Figure 3B), increasing C_t_ values especially of dilution steps with highly concentrated anti-Smad6 siRNA (Figure 4B). Still, the fluorescence signal of the lowest tested target concentrations of 1 pM remained distinguishable from NTCs.

#### 3.1.2. Quantification of Complexed siRNA

The major aim of this study was to show for the first time that RT-qPCR can be applied for sensitive and sequence-specific quantification of siRNA inside dendriplex preparations. Therefore, uncomplexed target siRNA shares in the dendriplex samples have to be quantified and compared to a dendriplex standard concentration dilution series. Dilution series containing anti-Smad6 siRNA dendriplexes (Figure 3C,D) instead of uncomplexed siRNA were carried out to show that the above-described assay is capable of quantifying uncomplexed target siRNA shares in the presence of dendriplexes and also to prove linearity of dilution series-derived standard curves. C_t_ values compared to reactions with uncomplexed target siRNA were increased (Figure 4), and hence sensitivity decreased. Therefore, the lowest analyzed concentration in experiments with dendriplexes was set to 31.62 pM instead of 1 pM and dilution steps were reduced from 10-fold to $\sqrt{10}$-fold. With further addition of randomized siRNA to these reactions (Figure 3D), no further sensitivity decrease was seen but the NTC again showed slightly increased fluorescence values in the final cycles of the PCR.

All dilution series performed were characterized by a high coefficient of determination (Table 1). Slopes and calculated PCR efficiencies were similar for each biological replicate of the same sample type with the exception of samples including both randomized siRNA and dendriplexes, which showed over 20% variability between the three biological replicates. Efficiencies were generally higher (and consequently regression line slopes smaller) in samples with randomized siRNA, probably caused by slight inhibitory effects. Due to slight fluorescence signal increases in NTC reactions in the case of randomized siRNA usage (Figure 3B,D), LOD and LOQ values were calculated with different formulas depending on the sample type. For dilution series with negative NTC, the following formulas were used:

LOD = 3.3 × (σ/s);(3)

LOQ = 10 × (σ/s),(4)

where σ is the standard deviation of the linear regression and s is the slope of the calibration curve [48,49]. Increase in NTCs (indicated by threshold line crossing) was considered using the approach by Wolfinger et al. [50], defining the LOQ (and also the LOD) as the lowest measured concentration that shows a C_t_ of at least 3.32 lower than the NTC. LOD and LOQ for samples including dendriplexes were furthermore limited by minimum concentrations tested in dilution series. Selection of concentrations for standard curve creation in this study was carried out in order to test reasonable siRNA concentrations for the drug delivery target application, and consequently there was no need for lower concentrated measurements for standard curve creation.

As clearly visible in Figure 4 and specified by coefficients of determination in Table 1, standard curves derived from dilution series as visualized in Figure 3 show a high degree of linearity, indicating good quantifiability over a wide range of target concentrations. C_t_ values are generally higher with dendriplexes present in the reaction: differences in comparison with samples containing only uncomplexed target siRNA are between six and ten cycles in case of reactions in absence of randomized siRNA (Figure 4A) and between four and seven cycles in case of reactions in the presence of randomized siRNA (Figure 4B). Technical triplicates expressed relatively low degrees of variance, as stated by the error bars (depicting standard deviations of technical triplicate mean values), indicating good intra-assay reproducibility. Highest levels of intra assay variation were seen in samples with low target concentrations as is common for qPCR [51,52]. Inter assay variance was examined by repeating PCR experiments on different days to generate separate biological replicates. Generally, differences between the reactions were more pronounced compared to intra assay measurements, which highlights the need for a separate dilution series as standard for each new experiment.

### 3.2. Influence of N/P Ratio on siRNA Accessibility

**Figure 5:**
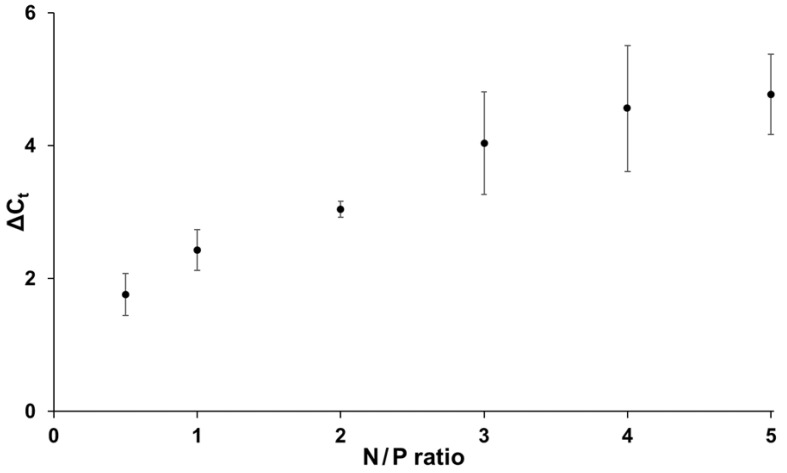
Differences in RT-qPCR C_t_ values between target siRNA samples with dendriplexes or uncomplexed target siRNA (ΔC_t_) at different N/P ratios. N/P ratio is defined as the amount of positive amine charges of PAMAM dendrimers and cell penetrating peptide LAH4-L1 divided by the negative phosphate charges of the siRNA in the sample volume. siRNA concentration was 10 nM (final PCR concentration in PCR) in all reactions. All reactions were performed three times in separate experiments (biological triplicates), each with technical triplicates. Arithmetic means are plotted and sample standard deviations indicated.

The N/P ratio is an important parameter affecting dendriplex properties such as siRNA complexation, accessibility and release profiles [24]. Compositions of dendriplexes with different N/P ratios were run in RT-qPCR experiments to evaluate effects of siRNA complexation on RT-qPCR results. In particular, quantifiability and reproducibility of PCR measurements as well as changing siRNA accessibility in samples with different degrees of complexation were analyzed. Therefore, samples with identical siRNA concentration but different N/P ratios ranging between 0.5 and 5 were prepared as described in the materials and methods section (Equation (1)) and C_t_ values were compared with equally concentrated uncomplexed target siRNA samples in a RT-qPCR (Figure 5). C_t_ increased when dendriplexes were present in samples, indicating either a higher degree of siRNA complexation and hence a lower accessibility of the siRNA for reverse transcription which ultimately resulted in decreased target amplification by polymerase, or sterical hindrance of reverse transcriptase and polymerase by the bulky PAMAM. Up to an N/P ratio of 3, increasing the PAMAM and LAH4-L1 concentration resulted in C_t_ increase. There are tendencies towards plateau formation at even higher C_t_ levels. Variance between individual measurements was relatively high when measuring samples with N/P ratios higher than 2, suggesting the existence of strong aggregation effects between components in highly concentrated dendriplex dispersions, leading to uncontrollable degrees of siRNA accessibility.

### 3.3. Effects of Complexation on siRNA Stability

**Figure 6:**
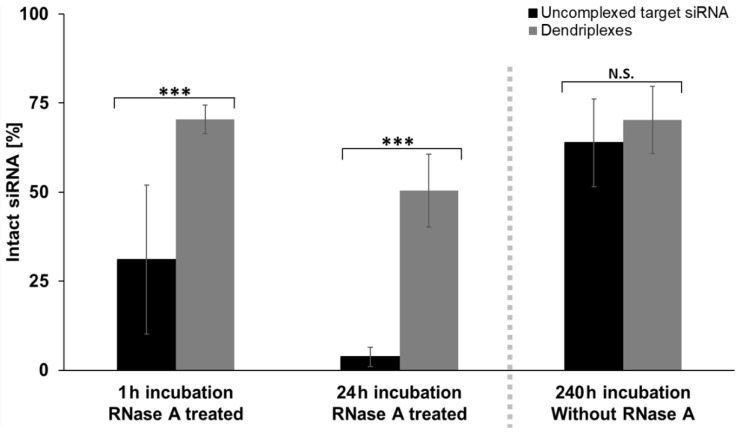
Influence of incubation time and RNase treatment on degradation of uncomplexed target siRNA or siRNA complexed in dendriplexes. Percentages of intact siRNA (in comparison to samples before incubation) after indicated incubation times were calculated as described in Section 2.4. RNase treatment was performed by adding 1 µg/mL (final concentration) bovine RNase A to the sample directly before starting incubation at 37 °C and 500 rpm. Concentration of siRNA at the start of each incubation was 100 nM, resulting in a theoretical concentration maximum of 10 nM in PCR reactions in case of no siRNA degradation. N/P ratio = 5 in all dendriplex samples. Three separate biological samples were prepared and each sample analyzed in PCR triplicate reactions for generation of each data bar. Significant differences between measurements with uncomplexed and complexed siRNA are indicated whenever identified by performing single factor variance analysis (ANOVA). N.S. *p* > 0.05; *** *p* ≤ 0.001.

One major benefit of using dendriplex carriers in drug compositions is seen in their protective impact on the active substance by shielding it from the environment. In case of siRNA complexation, protection from RNases has already been shown [18,26]. In order to confirm these findings and to test quantifying capabilities of the novel RT-qPCR assay, influence of complexation by PAMAM and LAH4-L1 on siRNA stability was tested in another RT-qPCR series. Therefore, samples with either uncomplexed target siRNA or siRNA dendriplexes at an N/P ratio of 5 were incubated for different time periods either with or without the presence of RNase A. Under RNase free conditions, siRNA remained stable over a long period (Figure 6, for calculation details see Section 2.4): After 10 days, more than half of the siRNA was still intact and no significant differences between uncomplexed (63.9 ± 12.3% intact) and complexed (70.3 ± 9.4% intact) siRNA samples were detected. RNase A treatment had a strong impact on the degradation process. Already after 1 h, the addition of 1 µg/mL RNase A resulted in degradation of high amounts of uncomplexed siRNA, with 31.1 ± 20.9% still intact, and after 24 h incubation, only traces (3.8 ± 2.7%) of initial siRNA were still intact. In contrast, RNase A mediated siRNA degradation in dendriplex samples was significantly slower, with 70.5 ± 4.0% intact siRNA after 1 h, and 50.4 ± 10.2% of siRNA still intact after one day of incubation. The degree of siRNA digestion by RNase A was highly variable in reactions with uncomplexed and hence unprotected siRNA after 1 h between biological replicates but not noticeably more variable between technical triplicate reactions compared to other performed PCR reactions.

## 4. Discussion

siRNA as a therapeutic has attracted interest with the approval of several drugs that have recently been successfully launched and many more in the pipeline awaiting final approval [3]. This development underscores the need for accurate and reliable methods capable of specifically quantifying untagged siRNA sequences and siRNA release from support structures in their native state, as well as measuring drug stability and progress of siRNA degradation processes for quality control. To address this need, we applied a previously developed PCR-based technique for siRNA quantification via stem-loop primers [23,47] to quantify siRNA release from dendriplex carriers and to gain knowledge about the concentration of complexed siRNA inside the complexes and overall complexation efficiency. For the first time, we showed that this method can be used to compare characteristics of dendriplexes with different N/P ratios and monitor siRNA degradation, reliably differentiating between target and non-target siRNA sequences. It was explicitly not an aim of this work to measure gene-silencing processes or to evaluate effects of the siRNA or carrier complexes on cells or other biological systems. Instead, it was intended to introduce a new application to characterize siRNA carrier systems for improved drug development regarding quality, efficiency and time needed for the development process. Other possible purposes of the assay are drug characterization and drug quality control enhancement. 

One central feature of a quantitative assay is its quantitation range. qPCR based assays usually need either an endogenous reference sequence or a target sequence quantification standard in every experiment for determination of parameters such as PCR efficiency, detection and quantification limits, and most importantly, target concentration in samples [53,54]. Establishing a protocol for reproducible creation of such calibration curves is essential. In this publication, anti-Smad6 siRNA sequence served as a target sequence. Measuring dilution series of uncomplexed target siRNA samples without the addition of randomized siRNA resulted in properly shaped amplification curves (Figure 3A) as expected from a qPCR assay without significant levels of inhibition [55,56]. Similar fluorescence intensity plateau heights with sigmoidally shaped amplification curves were reached and uniform C_t_ shifts expressed over the entire range of prepared dilutions. Since the applied synthetically produced siRNA samples do not contain common PCR-inhibiting substances which are often present in clinical samples [57], respective inhibitory effects would have been highly uncommon. To test competing siRNA in the assay for inhibitory effects and also to evaluate sequence specificity of the assay, other dilution series containing a fully randomized siRNA sequence (Figure 3B) were performed. Samples with highest target siRNA concentrations of these dilution series showed higher C_t_ values compared to dilution series without randomized siRNA and C_t_ distances between dilution steps were slightly decreased in the presence of randomized siRNA. This resulted in a lower slope of the calibration curve (Figure 4; Table 1) and hence in a higher apparent PCR efficiency, which in one case (Table 1) even exceeded 100%. These findings suggest a slight PCR inhibition caused by the randomized siRNA, which either blocks the reverse transcriptase from finding the target siRNA for amplification or interferes in stem-loop primer and target antisense siRNA duplex formation. The observation of “NTCs” showing minor increases of fluorescence intensities during the last 10 amplification cycles in dilution series with randomized siRNA should not be uncritically interpreted as false positive results, because randomized siRNA statistically bears a considerable amount of target siRNA molecules. It is also possible that few off-target sequences are detected false positively by the assay or the high concentration of nucleotides in solution or dispersions affects not only reverse transcriptase or polymerase activity, but also assay specificity. In the former case, mainly sequences with close homology towards the target sequence are suspected to be detected as has previously been shown or was assumed in other publications dealing with the topic of small RNA detection via RT-qPCR [58,59]. In both sample types (with or without randomized siRNA) quantification was possible in a concentration range of at least four orders of magnitude.

One of the main questions in this study was whether dilution series are still reliable and reproducible, when target siRNA is present in complexed form inside dendriplexes. Cell-penetrating peptides such as LAH4-L1 tend to form large aggregates [60,61,62]. This highly affects dilution series containing dendriplexes. In earlier publications, the dependency of dendrimer or cell-penetrating peptide aggregation on pH, electrostatic interactions and hydrophobic substrates has been discussed [63,64,65,66]. We observed that concentration irregularities of dilution steps vanished when the applied mastermix for PCR reactions was already present in the sample before dilution. It is worth noting that this procedure is essential for replication of the shown experiments in order to avoid calibration curve irregularities. Considering this, quality, uniformity and reproducibility of dilution steps with samples including dendriplexes (with target siRNA) was tested either with or without addition of randomized siRNA, as shown in Figure 3C,D. As can clearly be seen, C_t_ values were generally increased compared to those of dilution series with uncomplexed target siRNA. Thus, we can assume that significant amounts of siRNA must remain complexed even in case of partial heat-dependent decomplexation of the dendriplexes during reverse transcription of the siRNA. Distances between C_t_ values of adjacent dilution steps appear to be decreased on the first glance (comparisons of Figure 3A with Figure 3C; Figure 3B with Figure 3D) but concentration differences between dilution steps were reduced in dilution series with dendriplexes. This was done in order to improve discriminatory power of measurements, which due to higher LOD values (Table 1) was only possible in smaller concentration ranges. Despite these increased minimum LOD values, quantifiability was still ensured over at least 4 log units, when dendriplexes were present. Higher siRNA concentrations than 100 nM final PCR concentration (which equals 1 µM sample concentration) were not tested, because dendriplexes are usually applied in smaller concentrations [67,68,69] and show cytotoxic effects in high concentrations [70]. Hence, upper LOD and LOQ values were not determined. 

Aside from a generally higher C_t_ of samples with equally concentrated target siRNA and a resulting increase in LOD and LOQ values when using dendriplexes compared to samples without dendriplexes, no striking differences between PCR curves or standard curves were observed, regardless of whether randomized siRNA was additionally added or not. Therefore, it can be assumed that randomized siRNA does not cause decomplexation or lead to release of target siRNA from dendriplexes by substitution of the nucleotide sequences—at least not with this assay’s quantifiable amounts. When looking at the standard curves (Figure 4) it becomes clear, that biological replicates show higher deviations from each other than technical replicates. This behavior of increased measurement variability in independently prepared biological samples compared to semi-independent measurements by merely splitting samples during preparation steps is a commonly known issue in qPCR analysis [71]. These derivations are attributed to batch-to-batch differences in the course of the study and repetitive freezing and thawing of reagents. Hence, these errors could be prevented by using standardized solutions with known concentration. When utilizing the assay presented in this work for future studies, it is therefore highly recommended to apply single-use aliquots of the same batches of mastermix, oligonucleotides and target standard as discussed in previous works [71,72] to achieve the highest possible measurement accuracy and comparability of separate PCR reactions.

During the experimental phase for this work, it became evident that dendriplexes impede target siRNA amplification to a certain extend. Generally higher C_t_ values in dendriplex samples compared to likewise concentrated uncomplexed target siRNA samples led to the assumption that either dendriplexes interfere directly in the RT-qPCR process as inhibitors or sterically hinder siRNA from getting in contact with the stem-loop primer or reverse transcriptase. Comparison of dendriplex samples with different N/P ratios but the same siRNA concentration (Figure 5), which thus differed from each other only in their concentration of dendriplexes, revealed that dendriplexes do not directly inhibit PCR, since ever increasing N/P ratios would also have to lead to increased inhibition reaction which finally blocks the reaction entirely. Instead, as the dendriplex concentration increased, a plateau was presumably observed to be reached at an N/P ratio of about 3, from which the C_t_ stopped increasing any further, indicating the maximal binding capacity in this area. This strongly suggests that complexation caused shielding of siRNA from the stem-loop primer or reverse transcriptase and as a result, diminished accessibility of siRNA is the major reason for the C_t_ increase. In this case, the assay can also be utilized in complexation studies for defining amounts of uncomplexed siRNA inside dendriplex samples (and in turn complexation efficiency), general assessment of siRNA release from drugs or for estimation of N/P ratios of unknown samples. Furthermore, these results emphasize the need to select the right type of calibration curves: when samples with known N/P ratio but unknown target siRNA concentration are analyzed it might be more reasonable to use a dilution series with dendriplex standards with the same N/P ratio. On the other hand, measuring amounts of uncomplexed target siRNA in samples with unknown N/P ratio or complexation efficiency studies are more practical with dilution series of uncomplexed target siRNA. In summary, the application presented allows for siRNA quantification in dendriplex preparations. It does not directly quantify the total siRNA content in dendriplex samples, but rather measures it indirectly by determining the amount of uncomplexed siRNA available for PCR. This allows conclusions to be drawn about the actual total siRNA concentration if the N/P ratio is known. Additionally, our application enables the direct quantification of siRNA in samples without dendriplexes and the generation of drug release profiles if the N/P ratio is unknown.

Degradation of siRNA before reaching its cellular destination is a frequently discussed problem [73,74], not least because the nucleotides are exposed to RNases when they enter the bloodstream and therefore need to be protected. Aside from ensuring a constant release of siRNA, carriers also shield siRNA from molecules responsible for their degradation, as has already been shown for PAMAM dendrimers [68]. Our results (Figure 6) confirm these findings and show that the RT-qPCR based assay is able to detect siRNA digestion processes. Our assay application demonstrates its usability in future studies on the long-debated [75] questions, which RNases do digest small double stranded RNA, to what extent, and in what period of time [76,77,78], and what other factors [79] influence destruction of these nucleotides? Furthermore, we showed the applicability of the RT-qPCR based assay to the pharmaceutically and clinically relevant questions, which carriers or strategies can be used to protect siRNA on the way to its target site and for how long the drug is intact once entering the target site [27,80] as the assay solely detects and quantifies intact, non-degraded siRNA sequences.

Another finding of our study to examine siRNA stability is that siRNA release from dendriplexes does indeed not happen instantly, but over a long period of time in TLM medium; while uncomplexed siRNA samples were digested rapidly, RNase A did only digest approximately 30% of the applied target siRNA after 1 h and 50% after 24 h of dendriplex sample incubation (Figure 6), so we assume that a part of the siRNA must have been protected by dendriplexes and another part was released and freely available for RNase A during incubation. Possible explanations for high stability of not only complexed but also uncomplexed siRNA samples in absence of RNase A over 10 days are the use of stabilized Ambion^®^ In Vivo siRNA sequences (see Section 2.2) or a generally increased stability of small double stranded RNA sequences in a variety of conditions without RNase activity as assumed earlier [79].

In summary, it can be stated that RT-qPCR as means of the quantification of siRNA associated in dendriplex samples offers a combination of several advantages over previously used methods, such as differentiation between target and non-target sequences as well as between intact and degraded sequences, lack of the need to label target molecules, precise quantification while applying comparatively low sample volumes and concentrations compared to most current alternative siRNA quantification methods and the option to analyze more than one sequence at once in the same sample by multiplexing the assay. Follow-up studies to the presented findings could address siRNA release not only in dendriplex samples, but also in other drug carriers, such as liposomes or nanoparticles or even in matrices such as hydrogels. In addition, improved characterization of siRNA effects on human cell cultures by generating quantitative pathway data via multiplexed assays, enables simultaneously monitoring siRNA and its target mRNA. Apart from these more fundamental research-oriented suggestions for future studies, we propose to use and establish stem-loop RT-qPCR based assays in concrete applications with pharmaceutical benefit and drug development and production purpose, such as quality control checks. Furthermore, the use of this assay in studies quantifying degradation of differently protected siRNA (e.g., by locked nucleic acids [81], phosphorothioates or other common siRNA modifications [1]) in blood serum or even in cells could be highly valuable for the improvement of the stability of siRNA drugs and therefore enable advances in therapy.

## 5. Conclusions

In this study we showed that RT-qPCR can be applied to quantify siRNA in dendriplex preparations over a concentration range of at least 4 log units and with picomolar-ranged LODs and LOQs. This is sufficient for many queries related to siRNA-based drug development, such as drug characterization or quality control of manufactured drugs. We adapted the RT-qPCR based assay to be capable to differentiate between target and non-target sequences with a specificity, which is comparable to other existing RT-qPCR based assays for quantification of small RNA sequences. Aside from specificity, we furthermore demonstrated assay robustness by adding excess concentrations of non-target siRNA sequences, which was not interrupting the amplification process of this stem-loop based PCR technique to quantification-preventing degrees and showed only minor inhibitory effects. Moreover, our study demonstrated for the first time that dendriplexes do not inhibit the PCR. Availability of uncomplexed siRNA proportions in dendriplex preparations is directly quantifiable with the assay. It also allows assessment of complexation including estimation of complexation efficiency or comparison of formulations with varying N/P ratios and provides insights into the level of dendriplex-mediated siRNA protection from influences such as RNase degradation by quantifying siRNA integrity.

Our findings add the powerful, well established, fast and inexpensive tool of RT-qPCR to the list of suitable methods for qualitative and quantitative analysis of siRNA-containing preparations such as dendriplexes without the need of modifying the original chemical composition.

## Figures and Tables

**Table 1:** Statistical parameters, indicating intra- and inter-assay reproducibility and linearity of the assay under varying conditions. The threshold for determination of C_t_ values was kept constant at 0.03 RFU in all experiments. All dilution series were performed three times in separate experiments (biological triplicates), each with technical triplicates. Limit of detection and limit of quantification were defined as LOD = 3.3 × (standard deviation of linear regression/slope of the regression line) and LOQ = 10 × (standard deviation of linear regression/slope of the regression line) [48,49]. In case of NTC threshold line crossing, LOQ (and LOD) was calculated according to Wolfinger et al. [50]. Additionally, lowest experimentally measured concentrations were defined as minimum possible values for LOD and LOQ.

Randomized siRNA	Sample Type	Calibration Curve Replicate No	Coefficient of Determination	Slope	PCR Efficiency	LOD [pM]	LOQ [pM]
−	Uncomplexed target siRNA	1	0.99	−3.83	82.5%	4.1	70.3
		2	0.99	−3.82	82.6%	4.0	66.4
		3	0.988	−3.83	82.4%	4.5	96.6
+	Uncomplexed target siRNA	1	0.997	−3.31	100.4%	10.0	10.0
		2	0.998	−3.57	90.6%	10.0	10.0
		3	0.993	−3.49	93.5%	10.0	10.0
−	Dendriplexes	1	0.98	−4.47	67.5%	31.6	50.2
		2	0.98	−4.50	66.8%	31.6	51.3
		3	0.987	−4.78	61.9%	31.6	31.6
+	Dendriplexes	1	0.998	−3.52	92.4%	31.6	31.6
		2	0.995	−3.71	85.9%	31.6	31.6
		3	0.993	−3.15	107.8%	31.6	31.6
